# Multi-actuator light-controlled biological robots

**DOI:** 10.1063/5.0091507

**Published:** 2022-08-25

**Authors:** Jiaojiao Wang, Yueji Wang, Yongdeok Kim, Tianqi Yu, Rashid Bashir

**Affiliations:** 1 Department of Bioengineering, University of Illinois at Urbana-Champaign, Urbana, Illinois 61820, USA; 2 Holonyak Micro and Nanotechnology Laboratory, University of Illinois at Urbana-Champaign, Urbana, Illinois 61820, USA; 3 Department of Mechanical Science and Engineering, University of Illinois at Urbana-Champaign, Urbana, Illinois 61820, USA; 4 Department of Materials Science and Engineering, University of Illinois at Urbana-Champaign, Urbana, Illinois 61820, USA; 5 Carl R. Woese Institute of Genomic Biology, University of Illinois at Urbana-Champaign, Urbana, Illinois 61820, USA; 6 Department of Biomedical and Translational Sciences, Carle Illinois College of Medicine, University of Illinois at Urbana-Champaign, Urbana, Illinois 61820, USA

## Abstract

Biohybrid robots, composed of cellular actuators and synthetic scaffolds, have garnered much attention in recent years owing to the advantages provided by their biological components. In recent years, various forms of biohybrid robots have been developed that are capable of life-like movements, such as walking, swimming, and gripping. Specifically, for walking or crawling biorobots, there is a need for complex functionality and versatile and robust fabrication processes. Here, we designed and fabricated multi-actuator biohybrid walkers with multi-directional walking capabilities in response to noninvasive optical stimulation through a scalable modular biofabrication process. Our new fabrication approach provides a constant mechanical strain throughout the cellular differentiation and maturation process. This maximizes the myotube formation and alignment, limits passive bending, and produces higher active forces. These demonstrations of the new fabrication process and bioactuator designs can pave the way for advanced multi-cellular biohybrid robots and enhance our understanding of the emergent behaviors of these multi-cellular engineered living systems.

## I. INTRODUCTION

Biohybrid systems, utilizing the biological component to actuate synthetic scaffolds, have emerged as a reliable platform for understanding fundamental design rules and mimicking life-like motions. The living components in biohybrid systems offer unprecedented advantages over the conventional, rigid-bodied robots, such as self-assembly, self-healing, adaptation, sensitivity, and potential autonomy.^1–3^ Over the past decade, enabling technologies of 3D printing and tissue fabrication have advanced the development of biohybrid robots (biobots). Many forms of functional biohybrid robots are constructed to walk,^4–9^ swim,^10–14^ pump,^15^ grip,^16^ etc. In these biohybrid devices, the skeletal-muscle tissue functions as the sensor and the actuator, receiving the stimulation and converting energy to motion.^17–20^ The motion is generally displayed through free-standing flexible artificial scaffolds under external stimulation.

The development of various forms of functional biohybrid robots (biobots) has laid the foundation for understanding the governing biological principles, and exciting progress has been made toward building stronger and more robust devices.^8,9^ Yet, biobots are still far from envisioned functionalities and performances. The level of complexity, sophistication, and scale is still not comparable to living organisms.^13^ The key to recapitulate the microstructures and motion patterns of living organisms is the structural design and actuator performance.^20,21^ A significant step forward in the design and fabrication of such biohybrid robots requires a scalable and reliable fabrication process to enable a wide variety of biobot scaffolds and to produce high performance muscle actuators.

Here, with the goal of developing higher-order biohybrid robots, we present a new versatile approach to fabricate skeletal muscle-strip powered biobots of a variety of architectures. In this fabrication process, skeleton structures are assembled into the cell-gel injection mold prior to tissue seeding. Muscle actuators conform around the skeleton and mature in the assembled structure with the feet of the skeleton confined in the indentations of the mold. The formed biobot is contained by the mechanical constraint of the mold. The mold, in this case, functions not only as an injection template to retain the cell-gel solution but, more importantly, also as a static mechanical stimulus to reduce passive bending and maximize muscle force production. The modularity of this fabrication process offers wider customization and the flexibility to build large complex biohybrid robots with higher-order functionalities and of both strip^5^ or ring^6,7^ shaped actuators (Figs. S7 and S8). Combining the mechanical support imposed by the assembly with the enabling technology of optogenetics, we fabricated multi-actuator biohybrid walkers with bi-directional walking or multi-directional walking capabilities in response to noninvasive optical stimulation. We harnessed the advantage of optogenetics and precision stimulation enabled by enhanced spatial control^22^ to demonstrate light-guided multi-directional walking. These processes, designs, and demonstrations set the stage for developing dynamic robotic designs and advanced multicellular biointegrated systems.

## II. RESULTS AND DISCUSSION

### A. Characterization of optogenetic muscle bioactuator

**FIG. 1.**
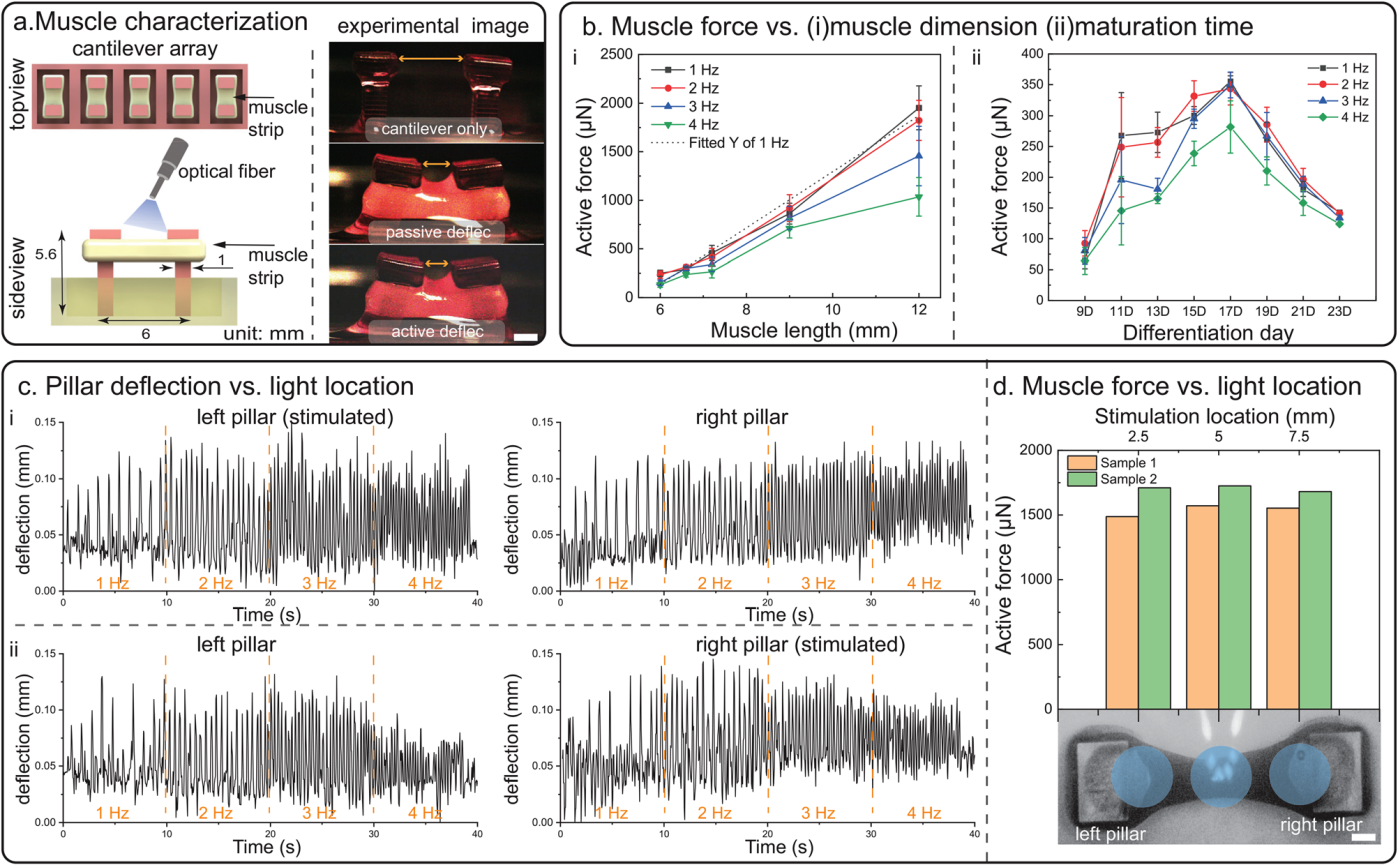
Characterization of optogenetic muscle strips. (a) Cantilever arrays are used for characterizing optogenetic muscle strips, and optical fiber is used to stimulate the muscle strips. Scale bar = 1 mm. (b) (i) Active force measured on muscle strips of different lengths on differentiation day 15 and linear fitting for 1 Hz frequency with R^2^ = 0.98, N ≥ 3. (ii) Active force of 6-mm muscle strips measured on different differentiation days. N = 3. (c) Pillar deflection data of the longest muscle strip—12 mm. (i) Deflection data of each pillar when only left pillar is being stimulated. (ii) Deflection data of each pillar when only right pillar is being stimulated. (d) Active force at 1 Hz of two 12-mm muscle strips induced by a laser pen at different stimulation locations along the muscle length. Light locations are indicated on the muscle strip. Scale bar = 1 mm.

Motivated by the goal of developing high-order biohybrid robots, we first performed a systematic characterization of optogenetic muscle actuators using cantilever arrays. Each characterization platform consists of five pairs of cantilever pillars with the bottom of the pillar fixed to the injection mold, in which 3D skeletal muscle strips are formed. To fully understand the muscle properties, we fabricated cantilever arrays with muscle strips of different lengths and monitored the pillar deflection every other day, starting at differentiation day 7. Both static resting tension and active contraction produced by the muscle strips were captured [Fig. 1(a)].

We fabricated muscle strip arrays of five sizes in lengths: 6, 6.6, 7.2, 9, and 12 mm (Fig. S1), four samples for each size, with the smallest size being the frequently used dimension in prior work.^6,7^ Muscle strips were stimulated with an optical fiber at multiple frequencies, 1, 2, 3, and 4 Hz. As expected, both passive tension (Fig. S2) and active forces calculated from the pillar deflection increase as the muscle length increases, with a maximum active force increase in eightfold from 6 to 12-mm samples [Fig. 1(b-i)]. As muscle tissue consists of a series of myotube fibers connected by the extracellular matrix (ECM) proteins,^8^ the muscle force is proportional to the number of myotubes and increases linearly with the increase in the muscle length. Next, we assessed the muscle force output of 6-mm samples from differentiation day 9 to day 23 [Fig. 1(b-ii)]. The analysis reveals that active forces continue increasing from 65±14, 93±21, 80±21, and 64±21 μN on day 9, corresponding to 1, 2, 3, and 4 Hz, respectively, to 355±9, 343±26, 349±21, and 282±42 μN on day 17 before the active forces drop to 143, 141, 134, and 124 μN on day 23. By differentiation day 23, only one sample showed trackable deflection. Optimal force output appears near differentiation day 17. Yet, biological samples can vary from batch to batch. The growth rate of the muscle force mentioned here can serve as a guide when determining the optimal time range and should not be the sole source to decide on the day of optimal performance.

**FIG. 2.**
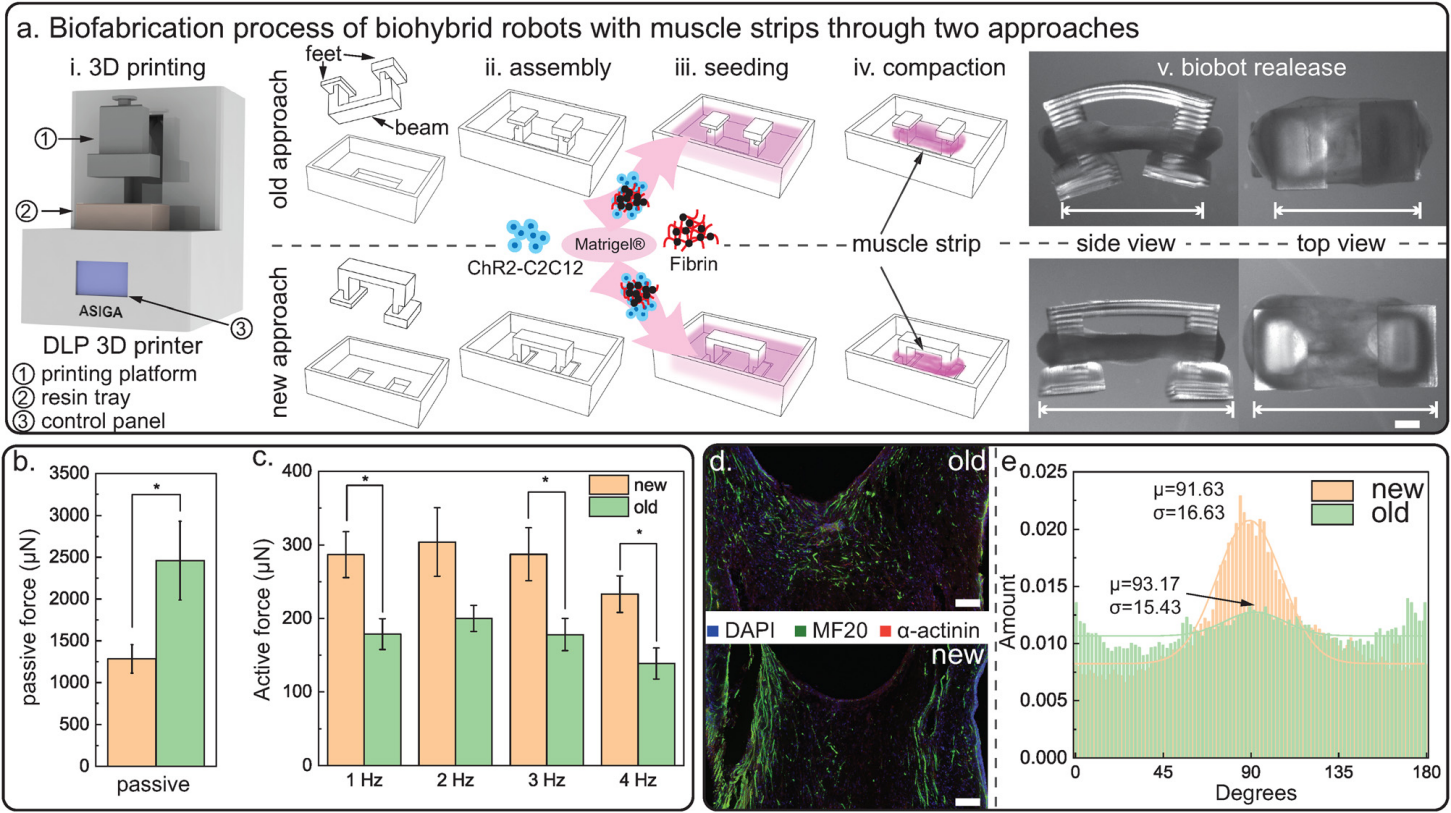
Fabrication of optogenetic single-muscle-strip biobots. (a) Biofabrication process of biohybrid robots with muscle strips through two different approaches: our previous approach^5^ and the newly developed modular scalable approach. (i) The mold and skeleton are printed with a DLP 3D printer. (ii) The skeleton is inserted in the mold either with feet facing up (old approach) or facing down (new approach). (iii) ChR2–C2C12 cells with ECM proteins are seeded in the skeleton-mold assembly. (iv) Cell-gel solution compacts after three days and forms single-muscle-strip biobots. (v) Biohybrid robots, with muscle strip wrapped around the skeleton, are released on the day of stimulation. Scale bar = 1 mm. (b) The passive force comparison of biobots from two fabrication approaches at differentiation day 10, N = 6, ^*^p < 0.05. (c) The active force comparison of biobots from two fabrication approaches at differentiation day 10, N = 6, ^*^p < 0.05. (d) Confocal imaging of muscle tissue slice expressing MF-20 and α-actinin. Scale bar = 200 *μ*m. (e) Histogram of myotube alignment of muscle strips from two different fabrication approaches.

Aiming to design biobots with multiple actuation sites, we then investigated the synchronicity and connectivity of the muscle actuator by locally stimulating the 12-mm muscle strip at different locations with a laser pen of a 2.6 mm spot size in diameter. However, even with the localized stimulation, all parts of the muscle contracted synchronously, and the induced contraction was observed along the entire muscle strip. With the laser pen stimulating on one pillar, the other pillar also displayed similar deflection [Fig. 1(c)]. The overall force output of the muscle samples was the same regardless of the stimulation location [Fig. 1(d)], and hence, we conclude that in these tissues and at these dimensions, multiple actuation sites cannot be achieved in one single muscle strip. This uniform response across the muscle length under localized optical stimulation is hypothesized to be a result of the connecting myotubes and the spot size covering the entire width of the tissue. As shown in the fluorescence imaging, the myotubes are connected by the ECM network and overlapping in the longitudinal directions (Fig. S3). The mean myotube width and distance between two adjacent myotubes were measured to be 11 and 6 μm, respectively, using Fiji ImageJ Software. Both myotube parameters are of the same magnitude as these reported in a detailed myotube morphology study.^23^ Given these myotube parameters and a spot size of 2.6 mm in diameter light source, the stimulation is always on more than one myotubes in the transverse direction. The induced muscle contraction can easily propagate from one end to the other through the overlapping myotubes. This observation presented the necessity of having multiple independent muscle actuators for multi-directional walking biobots instead of one connected muscle tissue with multiple actuation sites (Fig. S4). Exploring different sizes of light source for local stimulation was deemed as a less attractive path, beyond the scope of this investigation, and can be a future direction.

**FIG. 3.**
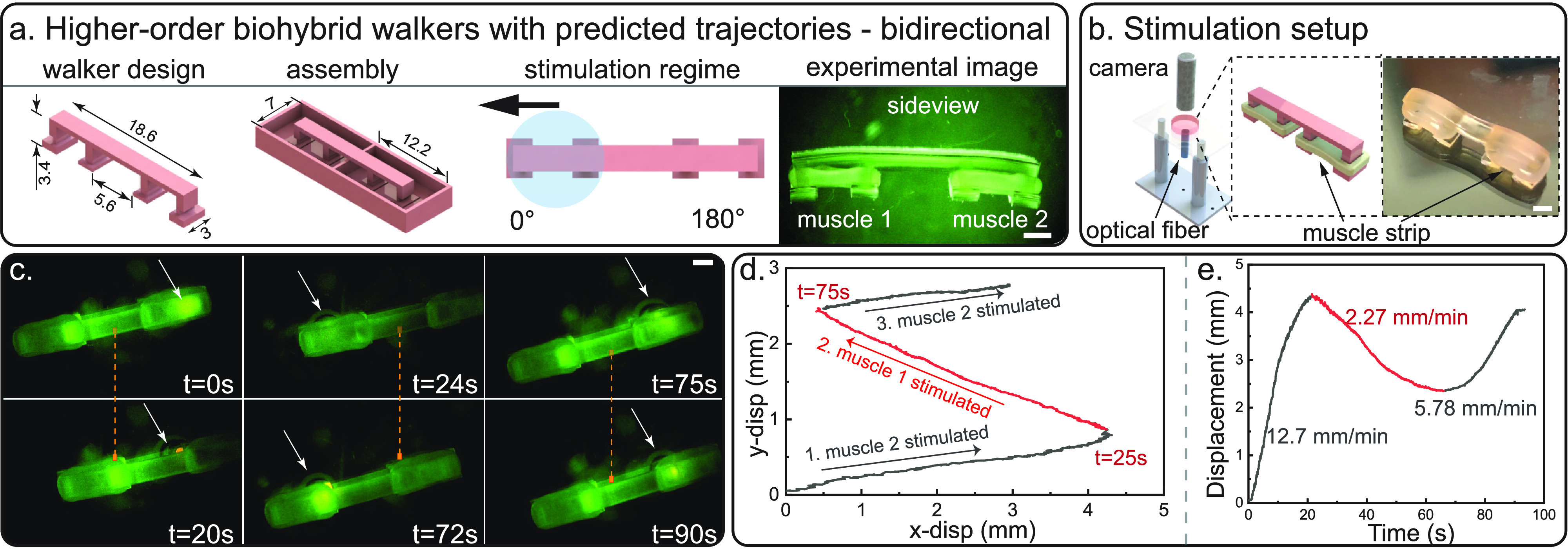
Demonstration of bi-directional walking (Movie S1). All scale bars = 2 mm. (a) Illustration of bi-directional walker: computer aided design (CAD) with major dimensions labeled, skeleton and mold assembly, stimulation regime with predicted walking direction, and experimental image. (b) Optical stimulation setup with optical fiber located under the two-muscle biohybrid walker. (c) Bi-directional walking is illustrated by three groups of still images from a representative walking video of differentiation day 15 (walking right–walking left–walking right). Optical fiber location is indicated by white arrows. See Fig. S12 for more bi-directional walking repeats. (d) Walking trajectory of the bi-directional biohybrid robot. (e) The displacement along the walking direction with velocities indicated on the plot.

**FIG. 4.**
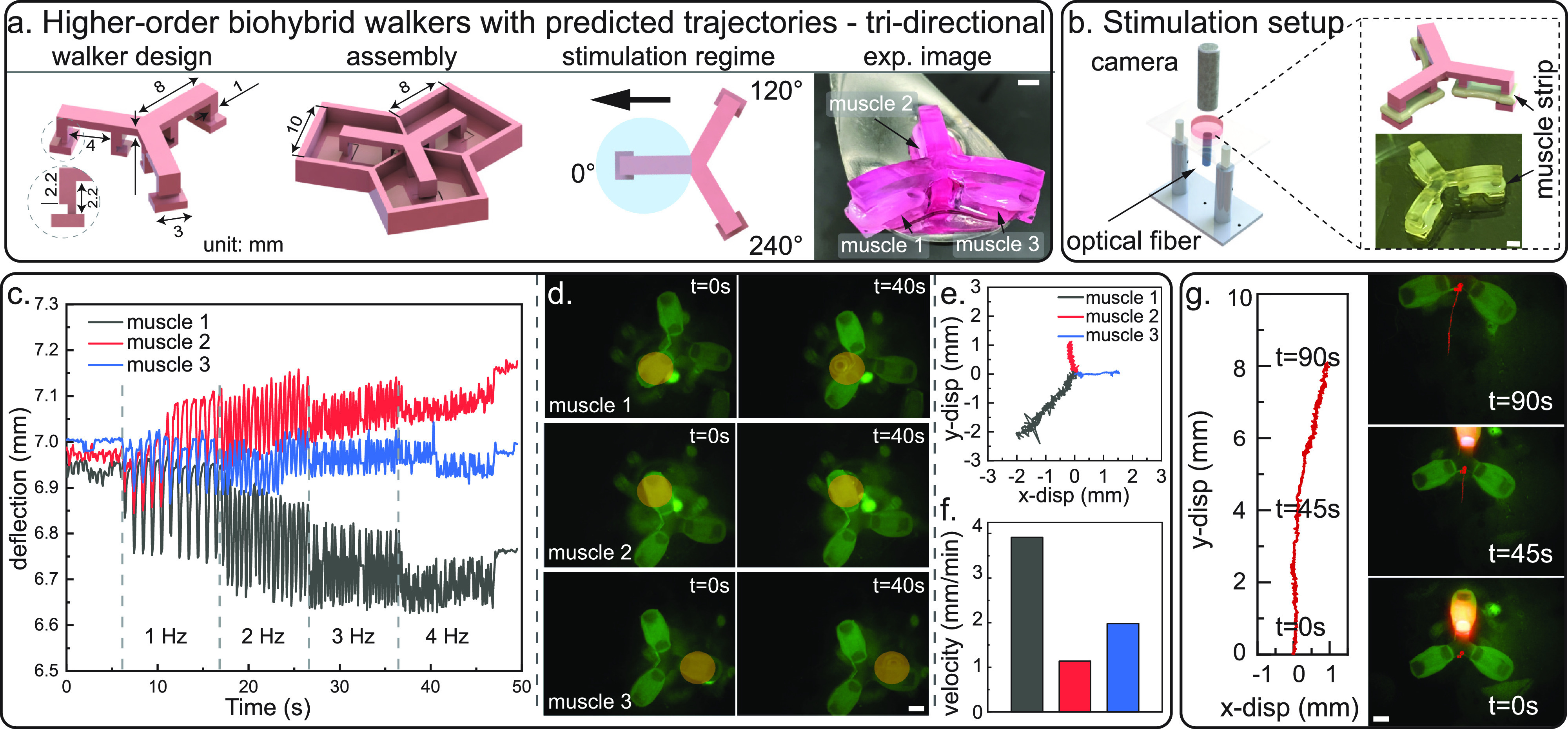
Demonstration of tri-directional walking (Movie S2). All scale bars = 2 mm. (a) Illustration of tri-directional walker: CAD design with major dimensions labeled, skeleton and mold assembly, stimulation regime with predicted walking direction, and experimental image. (b) Stimulation setup with optical fiber located under the three-muscle biohybrid walker. (c) Pillar deflection data of three muscle strips on a tri-directional biohybrid robot (recorded with feet facing up). (d) Tri-directional walking is illustrated by three groups of still images from a representative walking video of differentiation day 17. The optical fiber location is indicated by the orange circle. See Figs. S13–S15 for more tri-directional walking repeats. (e) Walking trajectories in all three muscle directions. (f) Walking velocities in three muscle directions. (g) Demonstration of continuous walking with optical fiber following the biohybrid robot walking path.

### B. A modular scalable fabrication approach for muscle-strip biobot

To design and fabricate functional multi-directional biohybrid walkers, we start by implementing a new fabrication approach that can reduce uncontrollable passive bending and maximize active muscle force production through the static mechanical stimulation. Static mechanical stretch during muscle maturation has shown to improve muscle performance by increasing its metabolic activity, cellular proliferation, and improving myotube alignment.^5,6,24^ In the previously published muscle-ring biobot studies, the static mechanical cue was imposed by the underlying glass coverslip, and the connecting beam of the biobot skeleton was kept tethered on the functionalized glass coverslip during differentiation to provide passive mechanical stimulation.^6,7^ Given the important role of static mechanical conditioning, we developed a modular scalable fabrication approach (Fig. 2) in which the injection mold kept the skeleton in place throughout the muscle maturation process. In this approach, the static mechanical cue was imposed by constraining the feet of the skeleton to the injection mold, thus reducing passive-tension-induced beam bending and providing a static mechanical stimulus.

In addition to the static mechanical cues, the modularity of this newly developed approach, enabled through the coupling of skeleton to mold, allows us for generating a wide range of biobot designs and the scalability in muscle actuator size and shape. The skeleton and the injection mold are designed in parallel for desired biobot architectures and muscle dimensions, before combining them to form the skeleton-mold assembly. Muscle actuator later forms around the skeleton-mold assembly. The modularity offered by the assembly step presents the possibility to scale and optimize the muscle actuator in size and shape through the modification of the skeleton and the mold.

Before implementing the new fabrication approach in the development of high-order biohybrid robots, we first fabricated single-muscle-strip biobots and compared them to single-muscle-strip biobots from the old fabrication process [Figs. 2(a) and S5] to demonstrate the merits of the newly developed approach. In the new fabrication approach, both feet of the skeleton are inserted into the matching indentations to form an assembled structure. The feet are bounded by the indentations on the mold; therefore, the connecting beam is maintained flat during the maturation process [Figs. 2(a) and S5]. Passive bending is limited by the mechanical constraint of the mold, whereas the beam is inserted into the mold with the feet facing up and free from any mechanical constraints in the old fabrication approach. During muscle differentiation, skeleton beam bends from spontaneous contraction of the muscle, and the feet come closer to each other [Figs. 2(a) and S5]. Starting at day 5, biobots from the old fabrication approach exhibit noticeably more intense spontaneous twitching than the biobots from the new approach. Consequently, the measured passive forces of biobots formed using the old approach ( 2460±471 μN) are higher than those from the new approach ( 1283±170 μN) [Fig. 2(b)]. The new approach with smaller passive forces shows great promise for fabricating functional biohybrid walkers as high passive forces are found to be detrimental to walking speed and directionality. A large passive force produces a more pronounced and sometimes uncontrollable bending of the backbone structure, which affects the contact angle of the feet and friction force between the leg and the walking surface. With a larger passive force, the biobot “tiptoes” on the petri dish surface. In our previous work, we noticed larger passive force indeed led to less consistent walking (in terms of directionality) and lower walking speed in both experimental settings and simulation.^9^

In addition to the difference in passive forces, we also measured and compared active forces induced by optical stimulation. At stimulation frequencies of 1, 2, 3, and 4 Hz, biobots produced by the new approach generated active forces of 289±31, 303±46, 287±36, and 233±24 μN, respectively, whereas biobots from the old fabrication approach produced active forces of 179±21, 199±18, 177±22, and 138±21 μN [Fig. 2(c)], lower at all four frequencies. The higher active force of the biobots from the new approach can be attributed to the static mechanical stimulation provided by the mold. Due to the confinement of the feet, the muscle strip wrapping around the feet is well-supported, and length of the muscle strip is maintained by two ends. Furthermore, we performed immunofluorescence staining on the muscle strips from both approaches to further inspect the distribution and organization of myotubes [Fig. 2(d)]. Myotube alignment was quantified using a fast Fourier transform (FFT) algorithm in Fiji ImageJ, and a higher degree of myotube alignment along the axis of tension was observed in the muscle strip from the new fabrication approach, a mean of 92 ° [Fig. 2(e)]. By contrast, the myotubes were less organized and more distorted in the muscle strip formed through the old approach [Fig. 2(e)]. We also performed RT-qPCR on the muscle strips to evaluate myogenic marker expression, and no significant fold changes were observed in gene expression of muscle strips from both approaches (Fig. S6). This could indicate that with both approaches functional and healthy muscle tissues were formed, but by comparing the muscle force output and biobot architecture from the two approaches in parallel, we conclude that the new modular and adaptive fabrication protocol introduced here offers substantial advantages for the development of complex biohybrid walkers.

### C. Demonstration of bi- and tri-directional locomotion

With the newly developed scalable fabrication approach and a better understanding of muscle actuator design rules, we scaled up in the skeleton design and proceeded to build multi-actuator biohybrid walkers. To enable multi-directionality, we fabricated and tested two next-generation biobot designs: two-muscle biobot with a 0°–180° muscle layout [Figs. 3(a) and S9] and three-muscle biobot with a 0°–120°–240° muscle layout [Figs. 4(a) and S10]. Each muscle actuator is independent from other actuators to allow for local stimulation and actuation.

To achieve unidirectional locomotion, biobots are generally designed to be asymmetric by having one leg shorter than the other, resulting in a net locomotion in the direction of the longer leg under external stimulation.^6,7^ With this as a guide, we took the advantage of the modularity of the new fabrication approach and formed overall symmetric biobots with two asymmetric biobots connected in series. Skeleton was assembled in the mold, and two muscle actuators were formed and differentiated in the assembled structure. Once matured, two-muscle biobot was deployed in the media and stimulated at 4 Hz by an optical fiber [Fig. 3(b)]. Bi-directional walking back and forth was indeed achieved by placing the optical fiber under different muscle actuators, with a walking velocity up to 13 mm/min [Fig. 3(e)], approximately one body length per min [Fig. 3(c)]. Furthermore, since the optical fiber can be moved freely, the locomotion direction can be easily alternated and guided by the light stimulus [Fig. 3(d)].

In addition to the bi-directional locomotion, we also explored more complex geometries, a tri-directional biobot with three independent muscle actuators [Fig. 4(a)]. Enabled by the same fabrication approach mentioned above and precise spatial control of optical stimulation [Fig. 4(b)], a three-muscle biobot was fabricated and showed consistent walking in three muscle directions with the walking speed ranging from 1 to 4 mm/min [Figs. 4(d)–4(f)]. The velocity difference in three directions is caused by muscle-to-muscle performance variation (Fig. S11). Under the same stimulation strategy, the peak-to-peak pillar deflection of three muscles varies by threefold [Fig. 4(c)]. Moreover, to prove the precise and enhanced control from optical stimulation, we utilized the optical fiber and guided a biobot for 90s until it walked out of the capture frame. Each time when the biobot walked out of the stimulation region, the optical fiber was manually moved to the actuating muscle to follow the biobot, providing consistent and precise stimulation [Fig. 4(g)]. This real-time guidance is not possible with remote electrical stimulation.^9^

Taken together, the newly developed modular fabrication approach and the controllability enabled by optical stimulation, we now have the capability to build biobots with much more complex geometries, for instance, eight-muscle biobots (Fig. S16), three-muscle T-shaped biobots (Movie S3), etc. The modularity, scalability, and mechanical constraint offered by the new approach provide the basis for complex skeleton geometries and muscle architectures. While we can produce devices reliably, nevertheless, it should be mentioned that yield of functional muscle actuators is still a limiting factor for large swarms of biohybrid robots. With the current fabrication approach, the yield of a functional 6-mm-single-muscle biobots is approximately 50% owing to variation in the cell source and muscle performance. With the increase in the number of actuators, the yield decreases. For example, to have two functional three-muscle biobots, we had to fabricated at least four batches with five biobots in each batch. Therefore, the difficulty and complexity of producing functional biobots still lie in engineering functional muscle tissues that are comparable to their biological counterparts. Improving the yield of high performance bioactuators with high consistency by further improving the biological processes and differentiation protocols^25,26^ and a detailed comparison to muscles developed *in vivo* must be pursued. Engineering approaches such as building redundancy in the formation of the muscle tissues and new designs could also be very important future directions.

## III. CONCLUSION

In this work, we introduced a new modular fabrication approach for skeletal-muscle-strip-driven biohybrid robots and characterized skeletal muscle strip in terms of growth rate, muscle length, and synchronicity within one muscle unit. Enabled by the new fabrication approach and a systematic understanding of muscle-strip actuators, we then fabricated biobots with two or three independent muscle strips to demonstrate bi-directional or tri-directional walking, respectively, under external optical stimulation. The reliability and controllability of the proposed fabrication approach have opened new possibilities for complex biobot designs, future on-board integration of electronics,^27^ and incorporating neuronal control^28–30^ and to subsequently broaden the applications in many potential fields.

## IV. METHODS

### A. Fabrication of mold and skeleton structures

The mold and skeleton structures of biobots and cantilever structures were designed in SolidWorks and exported as standard triangle language (STL) files for 3D printing. STL files were setup in the 3D-printing software (Asiga Composer) to specify printing parameters (layer thickness, exposure time, and CAD position on the printing platform). All structures were printed using a digital light processing (DLP) 3D printer (Asiga PICO2) at an exposure time of 3 s and a layer thickness of 0.2 mm. The resin solution was composed of 20% v/v polyethylene glycol diacrylate (PEGDA) 700 (Sigma-Aldrich) in de-ionized (DI) water with 0.1% w/v photo initiator, lithium phenyl-2,4,6-trimethylbenzoylphosphinate (LAP, Sigma-Aldrich) and 0.04% w/v Sunset Yellow dye (Sigma-Aldrich), a photoblocker to reduce light scattering effect.^9,31^

Printed structures were soaked in 10% bleach until the dye was gone, and then in 70% isopropanol at 4 °C. 70% isopropanol was changed to phosphate buffered saline (PBS) on the next day. The structures should remain in PBS at 4 °C for at least three days before seeding to minimize toxicity to cells.

On tissue seeding day, mold and skeleton structures were removed from PBS and placed in Petri dishes or six-well plates. The skeleton structure was picked up using a spatula and carefully placed into the mold with its feet aligning to the grooves. Extra PBS on the assembled structures were tapped away using Kimwipes, allowing for more precise alignment of the skeleton to the mold.

### B. Formation of biohybrid robots, biobots

C2C12 murine myoblasts were transfected to express a light-sensitive ion channel pro, Channelrhodopsin (ChR2[H134R]). ChR2–C2C12 cells were maintained in growth medium (GM) consisting of Dulbecco's modified Eagle medium (DMEM), 10% (vol/vol) fetal bovine serum (FBS) (Lonza), 1% (vol/vol) penicillin/streptomycin (Cellgro Mediatech), and 1% (vol/vol) L-glutamine (Thermo Fisher Scientific).^2^ Media were changed every other day, and cultures were kept in an incubator at 37 °C and 5% CO_2_.

Once cells reached ∼80% confluency, they were released from culture flasks using TrypLE (Gibco) and centrifuged at 1000 rpm for 5 min (RT) to form a cell pellet. The cell pellet was then resuspended and counted with a hemocytometer (Hausser Scientific Co.). With known cell concentration, the cell suspension was aliquoted into 15 ml conical tubes and centrifuged, with each tube containing a cell pellet of 3 × 10^6^ cells. Each cell pellet was then resuspended with 115 *μ*l GM+ containing 98% GM and 2% 6-aminocaproic acid (ACA, Sigma-Aldrich), following the addition of 6 *μ*l of 100 U/ml thrombin (Sigma-Aldrich), 90 *μ*l Matrigel (Corning), and 75 *μ*l fibrinogen (Sigma-Aldrich) at a concentration of 16 mg/ml. All cell pellets and ECM proteins were placed on ice during the seeding process to prevent coagulation. To form one muscle strip, 120 *μ*l of well-mixed cell-gel solution was quickly injected into the assembled structure. Assembled structures with cell-gel solution were placed in an incubator at 37 °C and 5% CO_2_ for 1.5 to 2 h to allow for further cross-linking. Warm GM+ was then added to the dish to submerge the seeded structures and changed every other day for 3 days.

Cell-gel mixture was compacted into a strip shape in the assembled structures after 3 days, and biobots were formed and kept in the molds. Media were then changed to the differentiation media (DM++) consisting of 98% differentiation medium (DM) supplemented with 2% ACA and 0.005% of insulin-like growth factor-I from mouse (IGF-1, Sigma Aldrich). DM consisted of DMEM supplemented with glucose, L-glutamine, and sodium pyruvate, 10% HI-HS (Fisher Scientific), 1% (vol/vol) penicillin/streptomycin, and 1% (vol/vol) L-glutamine. Biobots were kept in an incubator at 37 °C and 5% CO_2_, and DM++ were changed every other day.

### C. Optical stimulation

Biobots were released from the molds and optically stimulated to either acquire force data (sideview: feet facing the side) or test for walking (topview: feet facing down). To stimulate optogenetic muscle actuators, the LED module driver (465 nm wavelength) and optic fiber (Doric Lenses) were used with a power output of ∼2 mW/mm^2^ (corresponding to a spot diameter of 10 mm). For capturing cantilever pillar deflection, the LED fiber was placed above, directly on top of the muscle strip. Muscle strips were stimulated at 1, 2, 3, and 4 Hz (tens for each frequency) with a pulse width of 50 ms. To induce walking, the LED fiber was placed below the biobot [Fig. 3(b)]. Optical stimulation of biobots was conducted in warm DMEM without phenol red (Gibco). All deflection and walking data were recorded via a portable digital microscope (Dino-Lite). A red-light filter was utilized for better visualization and tracking.

To locally illuminate the long 12-mm muscle actuators [Fig. 2(d)], the laser diode module driver (Doric Lenses) with 450 nm of wavelength was used. The laser diode was attached to a 50 *μ*m core multimode NA 0.22 fiberoptic patchcord (Doric Lenses) to generate a laser beam with a diameter of 2.6 mm (power output:1.7 mW/mm^2^).

### D. Data analysis and force calculation

A tracking software (Tracker) was used to quantify the pillar deflection, and the pillar deflection data were then used for force calculation. Passive forces were determined using the passive displacement of the pillar before stimulation. Active forces were calculated based on the deflection induced by external stimulus.

#### 1. Cantilever force calculation

Muscle force on the cantilevers was determined using the following cantilever beam deflection equation (single load):


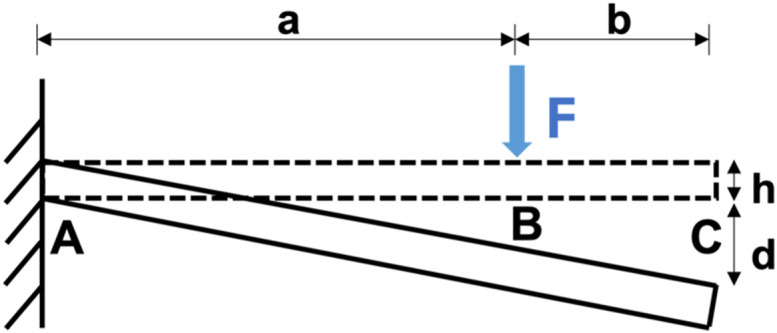

$$d=\frac{Fa^{3}(1+\frac{3b}{2a})}{3EI}.$$

The resulting force equation is as follows:

$$F=\frac{3EId}{a^{3}\left(1+\frac{3b}{2a}\right)},\;I=\frac{wh^{3}}{12}.$$

In this equation, F is the force exerted at B, a is length between A and B, b is length between B and C, I is moment of inertia of the pillar, E stands for Young's modulus (401 kPa),^9^ h is the pillar thickness, and w is the pillar width (into the paper, not shown on the schematic above).

#### 2. Single-muscle biobot force calculation

Muscle force equation for single-muscle biobots was derived from Euler–Bernoulli beam theory using angle of deflection (small-angle approximation). The detailed derivation steps can be found in a previous publication.^9^ The final equation used for calculating muscle force is as below:

$$F=\frac{\theta EI}{lL/2},\;\theta =\arcsin\left(\frac{d}{l_{\mathrm{leg}}}\right),$$

where L is the beam length, l is the moment arm (distance from beam to muscle location), θ is the angle of deflection, d is the measured displacement of one leg, and $l_{\mathrm{leg}}$ is the leg length.

### E. Cryosectioning and confocal imaging

Muscle tissues were removed from skeletons and fixed with 4% paraformaldehyde (Electron Microscopy Sciences) for 20 min at room temperature (RT). After washing with PBS, samples were treated sequentially with 10% and 20% sucrose solutions for 30 min and overnight with 30% sucrose in 4 °C. Tissues were embedded in optimal cutting temperature (OCT) compound (Tissue-Tek) and snap-frozen on dry ice before storing in −80 °C. The frozen tissues were sectioned with a 50 *μ*m layer thickness using a cryostat (Leica CM3050S) and placed onto the silane coated glass slides (Electron Microscopy Sciences). Sliced tissues were dried for 30 min at RT and rinsed with PBS to wash out OCT solution around the tissue slices. Samples were bordered with water repellent barriers, drawn with a PAP Pen (Abcam), and blocked with 1% bovine serum albumin (BSA) overnight in 4 °C. For immunostaining, mouse anti myosin heavy chain (MF-20) (ThermoFisher) and rabbit anti α-actinin (Abcam) were used as primary antibodies, with a 1:500 dilution ratio overnight in 4 °C. Then, the secondary antibodies, AlexaFluor-488 antimouse and AlexaFlour-647 antirabbit (ThermoFisher), were applied for 3 h at a 1:500 dilution ratio at RT to stain MF-20 and α-actinin antibodies, respectively. Then, samples were incubated with 4′,6-diamidino-2-phenylindole dihydrochloride (DAPI) of a 1:5000 dilution ratio overnight at 4 °C. Samples were washed three times with PBS and mounted with Vectashield Antifade Mounting Medium (Vector Laboratories) under sealed cover slips. The Multiphoton Confocal Microscope Zeiss 710 was used for imaging.

### F. Quantitative reverse transcription PCR analysis

Muscle rings were removed from biobot skeletons after force date collection and snap-frozen (5 min in liquid nitrogen). Samples were then stored at −80 °C and thawed on the day of RT-qPCR experiment. RNA was extracted using the RNeasy Plus RNA isolation kit (Qiagen), following the instructions provided. For quantitative PCR (qPCR), TaqMan fast advanced Master Mix, myogenic health PCR targets, and housekeeping gene, glyceraldehyde 3-phosphate dehydrogenase (GAPDH), were added to cDNA. PCR was performed on QuantStudio3: 2 min at 50 °C, 2 min at 95 °C, 40 cycles of 1 s at 95 °C and 20 s at 60 °C. Cycle threshold (Ct) values were obtained to calculate changes in expression level using by the 2−ΔΔCt method.^32^

## SUPPLEMENTARY MATERIAL

See the supplementary material for supplementary material figures on optogenetic muscle strip characterization, biofabrication of one-, two-, three-, and eight-muscle biobots, and additional walking data of bi-directional and tri-directional biobots and supplementary movies showing walking of bi-directional biobot (Movie S1), tri-directional biobot (Movie S2), and T-shape biobot (Movie S3).

## Data Availability

The data that support the findings of this study are available from the corresponding author upon reasonable request.
